# Machine Learning in the Parkinson’s disease smartwatch (PADS) dataset

**DOI:** 10.1038/s41531-023-00625-7

**Published:** 2024-01-05

**Authors:** Julian Varghese, Alexander Brenner, Michael Fujarski, Catharina Marie van Alen, Lucas Plagwitz, Tobias Warnecke

**Affiliations:** 1 Institute of Medical Informatics, University of Münster, Münster, Germany; 2 European Research Centre of Information Systems, University of Münster, Münster, Germany; 3 Department of Neurology and Neurorehabilitation, Klinikum Osnabrück - Academic teaching hospital of the University of Münster, Osnabrück, Germany

**Keywords:** Diagnostic markers, Databases, Parkinson's disease

## Abstract

The utilisation of smart devices, such as smartwatches and smartphones, in the field of movement disorders research has gained significant attention. However, the absence of a comprehensive dataset with movement data and clinical annotations, encompassing a wide range of movement disorders including Parkinson’s disease (PD) and its differential diagnoses (DD), presents a significant gap. The availability of such a dataset is crucial for the development of reliable machine learning (ML) models on smart devices, enabling the detection of diseases and monitoring of treatment efficacy in a home-based setting. We conducted a three-year cross-sectional study at a large tertiary care hospital. A multi-modal smartphone app integrated electronic questionnaires and smartwatch measures during an interactive assessment designed by neurologists to provoke subtle changes in movement pathologies. We captured over 5000 clinical assessment steps from 504 participants, including PD, DD, and healthy controls (HC). After age-matching, an integrative ML approach combining classical signal processing and advanced deep learning techniques was implemented and cross-validated. The models achieved an average balanced accuracy of 91.16% in the classification PD vs. HC, while PD vs. DD scored 72.42%. The numbers suggest promising performance while distinguishing similar disorders remains challenging. The extensive annotations, including details on demographics, medical history, symptoms, and movement steps, provide a comprehensive database to ML techniques and encourage further investigations into phenotypical biomarkers related to movement disorders.

## Introduction

Parkinson’s disease (PD) is a frequent neurodegenerative disorder with increasing incidence and worldwide burden^1,2^. The disease severely affects patients’ quality of life as the condition progresses. Similar to other movement disorders, PD is currently primarily diagnosed by clinical examination, which may be complemented by nuclear imaging. Typical symptoms of PD affect patients’ movement, such as rigidity, tremor, slowness, and difficulty of walking. Moreover, non-motor symptoms, such as depression, apathy, hallucinations, etc., can have severe negative impact on a patient’s well-being and are often reported as early indicators of the disease^3^. Although there is no neuroprotective or regenerative treatment to date, early diagnosis and treatment are particularly important to reduce burden and costs^4^. While PD symptoms are well known, individual disease appearance and progress is heterogeneous and hard to predict^5,6^. Technology-based systems have the potential to address these problems and aid in early disease recognition^7^. In this context, digital biomarkers could be established as objective measures that could be used in predictive or prognostic applications, as well as for assessing the disease severity. In this work, we focus on the diagnostic potential.

With the advancement of multi-sensor technology, previous works have already demonstrated promising PD classification based on various measures. A wide range of data modalities have been analysed, including hand movements, gait and balance, eye movements, and voice captures^8,9^. Most of these examples demonstrated high accuracy in the distinction of PD subjects from healthy controls (HC), yielding an important step towards clinical adaptation. The largest study to date provides more than 8000 unique subjects that performed the same task with comparable sensor recording using a smartphone app in a self-conducted home-based setting, however only with patient self-reported diagnosis^10^. In this context, we found analyses with reported accuracy of over 90% utilising voice captures or movement-based tasks^11,12^. Other studies utilised smartphones to track movement patterns throughout the day^13^ or recorded short self-conducted tasks, such as finger tapping or spiral drawing^14,15^. However, reports of high performance should be treated with caution, as many do not take into account confounding effects of working with unbalanced data, e.g., in terms of unbalanced age and gender distribution between cohorts^16,17^. Moreover, previous studies often lack control groups of similar movement disorders that would improve disease-specificity of the digital biomarkers. A classification model might perform well on differentiating PD from healthy, but it will misclassify other movement disorders and neurological conditions, such as Essential Tremor or Multiple Sclerosis, since the model learned general movement abnormalities, but not PD-specific features. In clinical reality, this is of high importance, as neurologists might have no prior knowledge of whether patients have PD or a similar movement disorder. While a subset of the researched studies included other movement disorders^12,18^, the controls were either restricted to a certain disease or did not have proper sample size of n > 100. Dataset size is crucial to validate generalisability and to reduce potential over-fitting. Further, many of the previous studies have applied rather passive monitoring, while interactive assessments would have the benefit of potentially provoking unseen subtle phenomena. For instance, PD tremor can show up under cognitive action or re-emerge when lifting and holding arms^19–21^. Different setups and datasets additionally introduce general comparability problems. In conclusion, a comprehensive public dataset utilising interactive clinical assessments and including a broad spectrum of PD and similar movement disorders has still been missing.

**Fig. 1:**
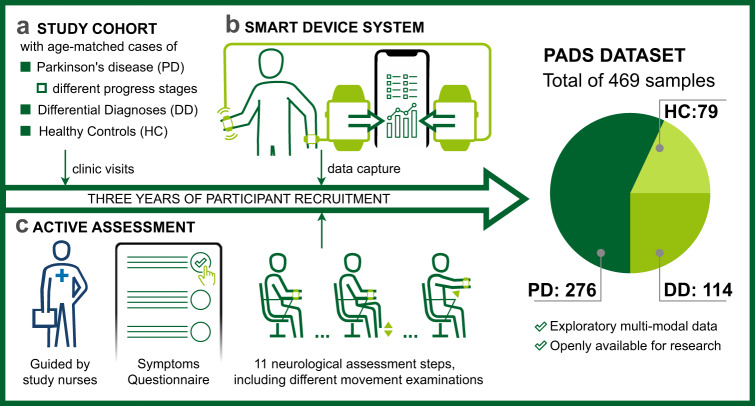
Study overview. The study cohort consists of cases of Parkinson’s disease (PD) in different progress states (according to the age at diagnosis), differential diagnoses (DD), and age-matched healthy controls (HC) **a**. Data capture was conducted via a custom application connecting two smartwatches and the smartphone, which we refer to as the Smart Device System **b**^22^. The active assessment was designed by expert neurologists and performed with two wrist-worn smartwatches **c**. The PADS (Parkinson’s Disease Smartwatch) dataset contains the entire sensor data from all assessment steps and details on symptoms, demographics, medical history, and diagnosis.

To approach these problems, a custom application integrating smart consumer devices and a centralised database, which we refer to as the smart device system (SDS), has been developed to analyse PD and similar movement disorders based on sensor recordings from an interactive assessment consisting of 11 neurological movement steps^22^. Smartwatches have demonstrated high potential as an instrument for precise quantification of movement in the context of PD analysis^23^. The validation of our experiment was conducted in the realm of geophysics, employing a comparative analysis with a gold-standard seismometer. The findings showcased a level of high precision of smartwatches in accurately determining acceleration amplitude and frequency, surpassing the perceptual capacities of the human visual perception applied in current clinical documentation^24^. The system is used as part of a compact assessment that was designed by movement disorder experts and takes approximately 15 min for each assessment. It includes patient’s self-completion of electronic questionnaires and a series of different movement-based assessment steps instructed by an interactive app. Two smartwatches were used to capture movements from both wrists simultaneously. Additionally, the records were complemented by extensive annotations on demographics, medical history, movement steps and PD non-motor symptoms (PDNMS). Data were collected and maintained by the central app. All assessments were accompanied by study nurses who checked compliance with the study protocol. Given this system, we have recorded 5544 clinical measurement steps of more than 500 participants in a three-year cross-sectional prospective study (summarised in Fig. 1). To the best of our knowledge, our resulting database is the largest collection of interactive movement measures from PD patients, similar movement disorders, and healthy controls using the integrated smartwatch and smartphone setup.

With this work, we publish the unique study data in a comprehensive collection, referred to as the Parkinson’s Disease Smartwatch (PADS) dataset. The data involves 469 individual cases after exclusion of erroneous samples and age matching. Information about usage and the link to the database can be derived from the data sharing statement. In addition, we performed an extensive evaluation of the dataset using state-of-the-art machine learning (ML) to analyse diagnostic performance. To ensure reproducibility^25^, the project scripts and settings are published in a code repository, see the code availability statement. Here, we have not only considered the distinction between PD and HC, but also the distinction between PD and differential diagnoses (DD). Both our app-based data acquisition system and our ML application are open source and allow for an integrative analysis that merges different data modalities. Overall, our work not only enables further research to boost diagnostic accuracy, but also provides a foundation that could be used for symptom monitoring and treatment adjustment, in which both non-motor and motor-symptoms can be captured more comprehensively by using patient reported questionnaires and sensors respectively.

## Results

### Data exploration

**Fig. 2:**
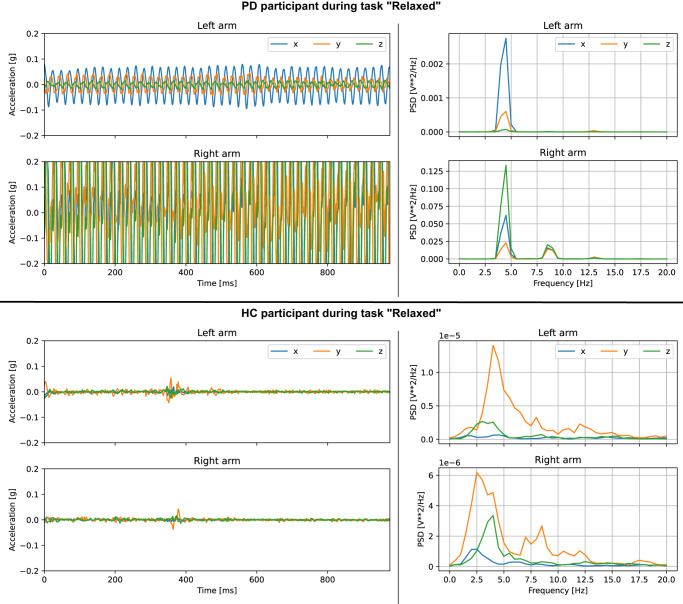
Data visualisation for a manually selected PD and a HC study participant. The top plot shows the acceleration curve of a PD participant during the “Relaxed” task, which was one of the 11 protocol-based assessment steps (see Table 4). The signal indicates a rhythmic tremor pattern that is more dominant on the right arm. The power spectral density (PSD) indicates a clear peak at around 4 Hz frequency. The bottom part shows the equivalent plots for a HC participant. The acceleration is closer to zero indicating that the arm was resting without tremor-like activity.

Besides the algorithmic analysis, the multi-modal data captures from our system can be visualised and controlled individually. Figure 2 compares the smartwatch-recorded acceleration of a manually selected PD participant with strong side-dominant tremor during the “Relaxed” task to a person from the HC cohort. The chosen example indicates clearly visible differences between the selected samples. In general, however, movement abnormalities are often subtle, especially in early cases of PD. Further, PD may implicate other movement symptoms besides tremor, such as gait and balance problems, rigidity and slowness. While ML analysis is used to automatically learn disease-specific characteristics, manual viewing of the recorded data can still be helpful for control reasons.

### Classification via machine learning

The study data was used for training and evaluation of Machine Learning models with nested cross-validation (CV). Based on the inner folds, a wide range of ML-based algorithms, including time-sensitive deep learning (DL) and classical signal processing were tested and a hyperparameter selection was performed using a grid search. The parameter space included the selection of input features, ML model and training parameters. Three ML options were applied to movement data and one for the simple and structured questionnaire data. Option A involved manually defined features in combination with classical ML, including Support-Vector Machines (SVMs) and feed-forward neural networks (NNs). Option B used automatic feature extraction based on the Bag-of-Symbolic-Fourier-Approximation-Symbols (BOSS) algorithm^26^. Option C applied automatic feature extraction via DL with XceptionTime^27^. Option D was applied to the questionnaire data utilising the decision-tree based classifier CatBoost^28^. Two classification tasks were analysed: (1) PD vs. HC and (2) PD vs. DD. We assume that the classification difficulty for a ML system is lower for the first task than for the second, since it only needs to detect non-healthy characteristics. PD vs. HC is presented primarily for comparative purposes. The second classification task requires more advanced feature analyses in order to distinguish movement disorders with very similar phenotypical characteristics.

The nested CV split the data into five (outer) test folds. Using balanced accuracy as reference metric, we report the scores per test fold using the best ML option selected from the optimisation based on the inner folds. Additionally, we report precision, recall, and the F1 measure. Table 1 summarises the results for the best-performing individual ML options.

**Table 1:** Classification results in each outer test fold after using the best setup (=input and classification pipeline) from the internal optimization fold.

Test fold	Input	Classification pipeline	Balanced accuracy	F1	Precision	Recall
**PD vs. HC**						
Smartwatch data only						
#1	Acceleration + Rotation	(B) SVM	69.55%	87.5%	85.96%	89.09%
#2	Acceleration + Rotation	(A) NN	76.31%	92.17%	88.33%	96.36%
#3	Rotation	(B) CatBoost	75.4%	91.23%	88.14%	94.55%
#4	Rotation	(B) SVM	87.9%	94.55%	94.55%	94.55%
#5	Acceleration	(B) SVM	85.77%	95.65%	93.22%	98.21%
Questionnaire only						
#1	NMS	(D) CatBoost	85.57%	89.32%	95.83%	85.57%
#2	NMS	(D) CatBoost	88.3%	92.45%	96.08%	88.3%
#3	NMS	(D) CatBoost	91.02%	95.41%	96.3%	91.02%
#4	NMS	(D) CatBoost	93.64%	93.2%	100.0%	93.64%
#5	NMS	(D) CatBoost	90.42%	92.45%	98.0%	90.42%
**PD vs. DD**						
Smartwatch data only						
#1	Rotation	(B) SVM	77.35%	68.29%	73.68%	63.64%
#2	Acceleration + Rotation	(B) SVM	74.98%	65.12%	70.0%	60.87%
#3	Acceleration	(A) NN	58.46%	43.14%	39.29%	47.83%
#4	Rotation	(B) SVM	68.46%	55.0%	64.71%	47.83%
#5	Rotation	(B) SVM	66.64%	52.38%	57.89%	47.83%
Questionnaire only						
#1	NMS	(D) CatBoost	74.68%	63.64%	63.64%	74.68%
#2	NMS	(D) CatBoost	60.47%	42.86%	47.37%	60.47%
#3	NMS	(D) CatBoost	68.62%	56.0%	51.85%	68.62%
#4	NMS	(D) CatBoost	68.06%	55.56%	48.39%	68.06%
#5	NMS	(D) CatBoost	67.0%	53.33%	54.55%	67.0%

Options A, B, and C were applied to smartwatch-data only, option D was applied to questionnaire data only. A: Manually defined features, shallow machine learning, B: Automatic feature extraction via classical signal processing, C: Automatic feature extraction via deep learning (not listed due to underperformance), D: Decision-tree based classifier.

Finally, Table 2 summarises average performance scores across folds and holds the results for classifier stacking, combining both questionnaire and movement information with different subsets of ML models. The movement-based classifiers achieved an average balanced accuracy of 78.99% for PD vs. HC and 69.18% for PD vs. DD. The classifiers based on the NMS questionnaires scored 89.79% for PD vs. HC and 67.77% for PD vs. DD. Our classifier stacking approach outperformed the respective classifications from movement and questionnaire data alone. While the score increased to 91.16% for the task PD vs. HC (1.37% change), the performance for PD vs. DD increased more significantly to 72.42% (3.24% change). Consequently, the numbers indicate that both data modalities add informational value to the system, allowing more accurate discrimination of PD from other movement disorders. Considering the costs of including both questionnaires and sensor records, the diagnostic performance gain may be considered marginal. However, it is worth noting that ML methods depend on the available data. In general, the prediction accuracy increases with larger sample sizes, as the training set becomes more diverse and the models learn to generalise. Comparable work on physiological signal data shows that this trend can persist up to a multiple of thousands of subjects^29^. As our sample size is by an order of magnitude smaller while having comparable input complexity, there is potential for further accuracy increases in future work.

**Table 2:** Average classification performance using cross-validation and classifier stacking (smartwatch + questionnaire).

Input	Test folds	Balanced accuracy	F1	Precision	Recall
**PD vs. HC**					
Smartwatch data	complete	78.99% (7.66%)	92.22% (3.18%)	90.04% (3.66%)	94.55% (3.41%)
	matched	76.39% (13.16%)	81.65% (11.55%)	72.94% (13.68%)	93.26% (7.73%)
Questionnaire	complete	89.79% (3.03%)	92.57% (2.18%)	97.24% (1.76%)	88.41% (3.97%)
	matched	87.67% (4.87%)	87.8% (4.35%)	90.65% (6.09%)	85.46% (6.16%)
Smartwatch + Questionnaire	complete	91.16% (4.92%)	94.62% (2.21%)	96.98% (2.49%)	92.4% (2.64%)
	matched	89.25% (5.65%)	89.77% (5.24%)	89.79% (8.99%)	90.11% (3.13%)
**PD vs. DD**					
Smartwatch data	complete	69.18% (7.45%)	56.79% (10.13%)	61.11% (13.57%)	53.6% (7.96%)
	matched	67.94% (12.68%)	62.11% (13.66%)	77.19% (20.7%)	52.55% (10.98%)
Questionnaire	complete	67.77% (5.06%)	54.28% (7.48%)	53.16% (6.51%)	56.21% (10.80%)
	matched	67.12% (7.11%)	61.5 (10.82%)	73.97 (12.93%)	53.48% (11.65%)
Smartwatch + Questionnaire	complete	72.42% (12.33%)	60.45% (16.7%)	54.92% (15.05%)	67.71% (19.45%)
	matched	69.56% (17.34%)	67.93% (19.84%)	70.36% (19.85%)	65.71% (19.93%)

Performance scores are given as mean (SD) across the five test folds. The classifiers used for the respective data modalities were chosen based on the optimization via internal cross-validation folds (see Table 1).

Although our dataset is balanced in terms of age distribution, there are still demographic differences in our study data. As our study cohort consists mainly of routine patient visits and concomitant control subjects, gender distribution is influenced by prevalence, which in the case of PD is known to be higher in men. To account for this imbalance, we extracted a subset of the complete dataset that was additionally matched by gender using a random under-sampling approach^16^. The subset consists of 234 samples (50% of the original sample size, more details in the dataset documents). In addition to the results for the regular test folds, we reported all performance measures for each test set and the corresponding matched subset. Table 2 compares the average scores across the matched test sets to the average scores across the complete test sets. The results for the matched test subsets showed a similar trend to the original results, where using multi-modal data input via classifier stacking increased classification accuracy. Given the classifier stacking setup, PD vs. HC was evaluated with a balanced accuracy of 89.25%, and PD vs. DD with 69.56%. These numbers lay within the standard deviation of the original scores, indicating robustness of the underlying models towards gender-specific biases.

### Feature importance analysis

**Fig. 3:**
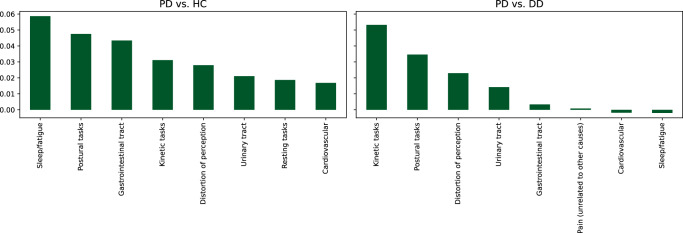
Grouped permutation feature importance applied to the best classification setup for the tasks PD vs. HC and PD vs. DD. Bar height corresponds to the average information gain in terms of balanced accuracy. Top feature groups are displayed.

We analysed information gain via grouped permutation importance to obtain greater insight into the decision process of our final model^30^. The high-dimensional feature vector was dissected into groups that represent certain attributes of the input. PDNMS questions were grouped by relevant domains (see dataset documents) and smartwatch-based features were grouped by assessment step categories (see Table 4). For each test fold, the input data, grouped according to the categorisation, were shuffled repeatedly and the change in (balanced) classification accuracy was recorded. Per feature group, the results were averaged across the random repeats and all test folds. That is, the analysis summarises the information gain resulting from the inclusion of specific questions on non-motor symptoms and the assessment steps recorded with the smartwatches. Figure 3 shows the top feature groups sorted by information gain for the two classification tasks. Both, the questionnaire and the movement data, improved diagnostic accuracy. The “Sleep/fatigue” questions rank highest in the classification task PD vs. HC, while this feature group has marginal influence for PD vs. DD. This indicates that the feature group is useful in distinguishing healthy samples from pathological ones, but may not be PD specific. For PD vs. DD “Kinetic tasks” achieved the highest information gain.

## Discussion

A smart consumer device setup with one smartphone and two paired smartwatches provides a powerful mobile system for the analyses of movement disorders. Given this setup, we conducted a three-year cross-sectional study to investigate phenotypical characteristics of PD and similar movement disorders. In previous work, the sensor precision of the utilised smartwatches was validated in using a high-precision shaker^24^. Within the scope of subtle tremor analysis, the deviations from the gold-standard setting were around 0.01 Hertz in frequency and in the range of 0.001 and 0.005 g in amplitude. Since human vision can hardly detect frequency changes of 0.1 to 1 Hertz and tremor amplitude of <0.01 g^24^, smartwatches can provide higher precision to assess tremor. In this work, we publish the full de-identified individual data to the scientific community and applied a combinatory ML pipeline to evaluate the diagnostic potential of the system.

When considering signal data from the smartwatches, the BOSS algorithm outperformed the other ML options in most cases. BOSS was applied at varying scales to extract different substructures of the signal while being relatively robust against noise and outliers. The advantage of automatised feature extraction is that it is not limited to predefined characteristics, such as only viewing tremor frequencies, but may consider other aspects of abnormality such as rigidity, or slowed and irregular movements. Although the use of DL has seen significant performance gains in the medical context, e.g., in the classification of ECG and EEG data, extensive amounts of training samples are required to robustly train such a system^31,32^. Although the self-reported non-motor symptoms are a subjective measure, they provide a more general picture of the patient’s condition that goes beyond the snapshot of the smartwatch recording. This informational value is also reflected in the classification scores, where for PD vs. HC the questionnaire-based classifier outperformed the classifiers applied to signal data. For PD vs. DD the movement-based classifier was slightly more accurate on average.

Furthermore, we observed that when combined, the two data modalities—the smartwatch recordings and the PDNMS questions—were relevant for classification and increased performance scores. The classifier stacking outperformed classifications using only smartwatch data or questionnaire answers, in particular for the task PD vs. DD. These results indicate that the usage of different data modalities aid in characterising PD more precisely when comparing it to similar disorders. The influence of the feature categories was further illustrated by our analysis of feature importance.

Our presented ML experiments yield promising results in the classification of PD vs. HC with a balanced accuracy of 91.16% and were based on a large participant population. While the classification of PD vs. DD achieved reasonably well performance with balanced accuracy of 72.42%, the score still lags behind the aforementioned classification setting. The latter setup, however, more closely resembles clinical reality. By additionally computing the classification scores on gender-matched subsets of the test folds, we have shown that the classification results were not strongly biased by gender imbalance. Given these subsets, PD vs. HC was evaluated with 89.25% balanced accuracy and PD vs. DD with 69.56%. The results show the overall diagnostic potential of the system. To investigate, whether it could be more accurate than clinicians, however, would depend on the clinical experience and infrastructure of the region, and require follow-up work with a different study design.

Overall, it should be noted that the standard deviation in our evaluation was relatively high, e.g., up to 12.33% for the balanced accuracy in the distinction of PD and DD cases, which is also due to the limited test set sizes. Large feature spaces in some setups may also have caused overfitting to training data, which should further be regularised in future work. Another limitation of our study is that it was conducted at a single site. Therefore, the risk of potential bias due to specific site population, examination setting, or target diagnoses cannot be fully ruled out. Still, we believe that the study data provides a suitable representation, as participants were recruited from a large tertiary care centre and include various types and stages of PD and other movement disorders. This inclusion is highly important for adapting well to clinical reality. It should be noted that clinical information on progress states, such as Hoehn and Yahr scales^33^, are not available in this dataset, but could be further extracted from discharge letters on request. Our control cohorts were age-matched to the PD cohort to best represent the risk group. The PD cohort is the main cohort of our study and includes more male patients, which corresponds to the known gender distribution of PD in the world population^1^. Although we reduced the bias due to general age-related changes, the male gender in our data set is still underrepresented in the control groups compared to the PD cohort. While we have accounted for this problem by using sample weighting and evaluating on additional gender-matched test subsets, systematic causal analysis should be conducted in future studies to rule out confounding effects^17^. Another limitation of this study is that our results are based on a one-time in-clinic assessment. PD symptoms can exhibit temporal variability, and the clinical observations on the day of recording may not necessarily reflect the patient’s condition throughout their daily life. Further, the data quality achieved in this study was facilitated by the presence of the study nurse who could oversee and control the recording process. In e.g., a home-based setting, where such control may be less feasible, there is a potential for decreased data quality. For future evaluations, it is therefore advisable to provide for automatic quality control methods, e.g., checking for unexpected behaviour that does not conform to the protocol^34^.

To the best of our knowledge, we contribute the largest collection of smartwatch recordings from an interactive assessment including PD and DD classes so far. Along with movement data, the PADS dataset is supplemented with extensive contextual information, such as disease details from various movement disorders, patient demographics, medical history, and non-motor symptoms. While we have shown an exemplary approach to evaluate the potential for diagnostic biomarkers utilising advanced ML, it provides a reference dataset to the ML research community for developing and comparing novel algorithms in the field of movement disorders. The presented system, its app and analysis tools, can be re-used (e.g., with simple assessment steps from the protocol of other observational studies) and fine-tuned with the existing data. With increasing advances in transfer learning^35^, the dataset has the potential to provide an essential data foundation for the development of future sensor driven systems for movement disorders. Besides classification, follow-up work could further address disease progress and symptom severity prediction, enabling unbiased, objective assessment^36^.

In this work, we comprehensively evaluated the informational value of a smart device-based system with interactive neurological assessment. In conclusion, our findings suggest that a system like this, combined with advanced ML, has the potential to aid in clinical routine and advance research related to movement disorders. Since the entire system is based on consumer-grade devices, it could potentially be used for upcoming home-based assessments and the extracted features can serve as a disease monitoring tool. The app gives step-by-step instructions on how to perform the assessment so that patients could use it on their own. While we have proposed a setup with two smartwatches, the system could be further simplified, e.g., with only one smartwatch, or extended with other sensor recordings and data captures. In this context it is noteworthy that the number and availability of low-cost sensors and consumer devices such as smartwatches that integrate them is increasing rapidly^37^. In the future, we will include new data modalities, such as voice captures, spiral drawings, and finger-tapping tasks, which have already been pre-tested on a small sample size^38^. Lastly, we plan to expand our study cohort and involve long-term progress monitoring, so that the presented system and potential biomarkers could not only be evaluated for diagnostic, but also predictive relevance (e.g., trend biomarker).

## Methods

### Study data

The cross-sectional prospective study recruited participants from the year 2018 until 2021. It has been registered on ClinicalTrials.gov with the ID NCT03638479 (registration date 20-08-2018) and approved by the ethical board of the University of Münster and the physician’s chamber of Westphalia-Lippe (Reference number: 2018-328-f-S). All participants gave written informed consent to take part. The study design and the protocol have been published previously^22^. All sessions were recorded upon routine visits to the outpatient clinic of movement disorders at the University Hospital Münster in Germany, which is one of the largest PD assessment centres in Germany. Three participant groups were recorded: (1) PD, including a broad range of ages and onset of diagnoses, (2) differential diagnoses (DD), including Essential Tremor, atypical Parkinsonism like Progressive supranuclear palsy (PSP) or multiple system atrophy (MSA), secondary causes of Parkinsonism, and Multiple Sclerosis, and (3) healthy controls (HC). The control groups were age-matched to the PD cohort to allow a fair comparison.

**Fig. 4:**
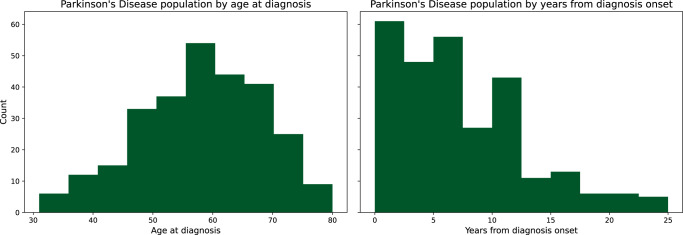
Overview of the Parkinson’s group. The left histogram shows the sample count by patient’s age at diagnosis. The right shows the sample count by years since first diagnosis at the time of inclusion in the study.

All diagnoses were established by board-certified neurologists and additionally reviewed by one senior movement disorder expert. As a routine standard, advanced DaT (Dopamine Transporter) scan imaging is ordered in cases of uncertainties. Currently, there is no 100% accurate diagnostic marker for PD, only the existence of pathological proteins after brain biopsy. Thus, we cannot guaranty 100% accuracy of the labels, but at least the most advanced state-of-the art clinical and technical procedure, which provides highest diagnostic accuracy when compared to neurologist practices. Originally, 504 individual participants were recorded. Quality, correctness, and completeness of diagnoses and signal data were checked throughout the study and after completion. Erroneous records and samples with missing data were removed (two samples dropped out due to missing data; from the youngest controls 33 were randomly removed as they did not match the age distribution of PD patients). At the end of 3-year recruitment, all included cases were checked via patient record review by a senior PD specialist resulting in eight subject labels to be corrected). The resulting participants’ population statistics are listed in Table 3. In addition to summarising the three aforementioned groups used for ML, Table 3 also provides differential diagnosis subgroups. Figure 4 gives an overview of the spectrum of PD patients in the dataset. Further information on differential diagnoses and demographics is provided for each sample in the dataset documents (see data availability statement).

**Table 3:** Participant samples including Parkinson’s disease (PD), healthy controls (HC) and differential diagnosis (DD).

Class	Participant group	Sample size (male, female)	Age, years (std)	Age at diagnosis, years (std)	Height, cm (std)	Weight, kg (std)
**PD**	**Parkinson’s disease**	276 (195, 81)	65.4 (9.6)	58.26 (10.62)	175.71 (9.77)	83.78 (18.1)
**HC**	**Healthy controls**	79 (29, 50)	62.9 (12.5)	—	170.48 (15.63)	78.75 (16.72)
**DD**	**Differential diagnoses**	114 (57, 57)	62.4 (11.5)	52.96 (16.52)	173.04 (8.73)	79.54 (17.84)
	Atypical Parkinsonism	15 (8, 7)	66.0 (7.96)	62.6 (8.21)	168.47 (8.1)	79.13 (18.64)
	Essential Tremor	28 (18, 10)	66.18 (10.84)	46.96 (22.89)	173.36 (9.6)	84.68 (21.88)
	Multiple Sclerosis	11 (7, 4)	54.09 (7.92)	43.82 (13.04)	178.36 (10.69)	83.55 (16.18)
	Other	60 (24, 36)	61.32 (12.32)	55.02 (13.32)	173.07 (7.64)	76.52 (15.45)

Participant examinations consisted of two parts: (1) an electronic questionnaire and (2) active movement-based assessment, all guided by the central app on the smartphone. Each recording session started with the questionnaire that was self-completed by the participants via the smartphone app. This questionnaire comprised two steps. First, answers about medical history were captured, including information about age, height, weight, kinship with PD, effect of alcohol on tremor, and medication (further details provided in the original study design by Varghese et al.^22^). The second component included 30 yes/no answers about PD specific non-motor symptoms (PDNMS) based on the PDNMS questionnaire from the International Parkinson and Movement Disorder Society^39^. The questions cover a wide spectrum of domains, ranging from cognitive problems, such as hallucinations, depression, apathy, and anxiety, to sexual dysfunction, sleep disorders, bowel problems, and sailorrhea. We previously evaluated ML-based classification using questionnaire data only and found high informational value utilising the 30 PDNMS questions^40^. A feature importance analysis showed that the classification accuracy did not increase by including additional personal information, such as age, sex, height, and weight. Therefore, we used the 30 PDNMS answers in this work and excluded other personal data. Clearly, the collected medication data was acquired with the intention of enhancing our dataset for future research purposes, but it will not be utilised for ML since it already provides indicative information regarding the diagnosis. Despite the subjective nature of patient-reported outcomes, they provide valuable and long-term patient perceived insights. These complement the sensor-based approach, which only captures a temporary status during the assessment and therefore might miss disease characteristics of patients in off-state.

After completing the questionnaire, participants continued with movement examination steps. These were instructed by the smartphone app and guided by a study nurse who ensured adherence to the protocol. The assessment steps were designed by movement disorder experts with the primary aim to establish a simple-to-follow examination and to capture the most relevant movement characteristics. For data capture, each participant wore two smartwatches (Apple Watch Series 4), one on each wrist, while seated in an armchair. During each assessment step, the smartwatches synchronously recorded acceleration and rotation data on three spatial axes (x, y, z) with a sampling frequency of 100 Hertz. In total, 11 interactive assessment steps were performed, three of which took 20 s and the rest 10 s, which are summarised in Table 4. Data capturing were handled by the central app, which connected all devices and the study database. For the ML analysis, the 20-second recordings were cut into two parts, yielding 14 time series of 10 s per participant. With this setup we achieved 168 channels of time series data for each participant (14 tasks × 2 arms × 2 sensors × 3 axes = 168). In preliminary experiments, we assessed the acceleration sensor on its own and found high informational value. Here, we evaluated three experimental setups, (1) using acceleration only, (2) using rotation only, and (3) using data from both sensors to account for all data. This selection of the input source is treated as an own hyperparameter in the ML evaluation. Based on previous data analysis, we removed three assessment steps that gave no additional value to the classification: step three (“lift and hold”), step five (“point finger”), and step eight (“touch index”)^41^. The first 0.5 s of every time series were removed to cut out smartwatch vibration that notified about the start of each recording. Further, we pre-processed the acceleration data for ML analysis to account for the influence of the Earth’s gravitational field. Gravitational orientation changes were corrected with the help of l1-trend-filtering, which functions as piece-wise linear smoothing. Similar to Little et al. we subtracted the estimated low-frequency gravitational trend from the signal data to remove the offset from the physiological data^42^.

**Table 4:** Smartwatch-based assessment steps.

Steps	Durations	Description	Task category
1a	20	Resting with closed eyes while sitting, positioning standardised to Zhang et al.^41^.	Resting
1b	20	Resting while patient is calculating serial sevens.	Resting
2	10	Lift and extend arms according to Zhang et al.^41^.	Postural
3	10	Remain arms lifted.	Postural
4	10	Hold one-kilogram weight in each hand for 5 s. Start with the right hand. Then, have the arm rested again as in 1a.	Postural
5	10	Point index finger to the examiners lifted hand. Start with right index, then left Repeat the movement.	Kinetic
6	10	Drink from glass. Grasp an empty glass as if drinking from it. Start with the right hand. Then repeat with the left hand	Kinetic
7	10	Cross and extend both arms.	Kinetic
8	10	Bring both index fingers to each other.	
9	10	Tap own nose with index finger. Start with the right, then with left index. Then extend the arms.	Kinetic
10	20	Entrainment. The examiner stomps on the ground, setting the pace. Start stomping with the right foot according to the pace. Leave the arms extended during the movement. Repeat this with the left foot.	Postural

Durations are reported in seconds (s).

The dataset holding all records is organised in JSON text files, readable and editable in any editor. While there is not yet an established format standard for metadata of medical time series derived from wearable sensors, we follow the specification by Claes et al.^43^, which refers to the similar data as in our study. Further details on data usage can be derived from the data availability statement.

### Machine learning

We determined a fixed randomisation seed for a 5-fold CV to increase fairness in the evaluation and to ensure reproducibility. Scores were reported as mean values over the resulting five test sets. While CV ensures strict and systematic separation of training and testing, we further point out that assessment steps of the same individual are bound to one data sample. As a result, no sub-time-series of one individual are mixed in training and testing, which could cause identity confounding^44^. Moreover, to increase generalisation and counteract overfitting, hyperparameters, including the selection of classification models and input signal, were conducted via an additional inner 5-fold CV per training iteration using a grid-search. This approach is referred to as nested CV. Implementation details and tested hyperparameters can be derived from the code repository. We measure and compare performance based on balanced accuracy to guarantee more representative and stable performance scores in case of class imbalances. In addition, we evaluated precision, recall, and the F1 measure. Three different options were tested on the smartwatch records (options A, B, and C). These range from the common approach of using manually defined features in combination with classical classifiers, such as SVMs, to specialised signal processing, and DL for time series. Training and testing were performed using the Python libraries scikit-learn (version 1.1.0)^45^, pytorch (version 1.11.0)^46^, and skorch (version 0.11.0)^47^.

Option A involves manually defined features and shallow ML. In previous work, we developed a set of relevant features derived from acceleration data based on an intermediate state of the study^24^. Based on the prior experiments, we focused on features using statistics and frequency measures of the input signal. Feature computation was based on the following steps: (1) The power spectral density was computed in discrete 1 Hertz steps using Welch’s method^48^. Values for 0 Hertz or above 19 Hertz were discarded, resulting in 19 frequency features per channel that were scaled by logarithm afterwards. (2) Each time series was split into four segments of equal length. For each segment, the standard deviation, the maximum absolute amplitude and the sum of absolute energy was computed, resulting in 12 features per channel. In total, each channel was transformed into a feature vector with 31 elements. Finally, all channels were concatenated. The manually derived feature representation was passed into a set of different ML classifiers to compare their performance. We have chosen to test three different options, the SVM, CatBoost, and a fully connected feed-forward NN.

Option B is based on automatic feature extraction via classical signal processing. Using the Symbolic Fourier Approximation (SFA) method, the Bag-of-SFA-Symbols (BOSS) algorithm extracts substructures of the signal^26^. We used three sliding windows with a hierarchical sizing of 20, 40, and 80 sample points length to extract information from different scales of the signal. Per window length, one BOSS transformer was applied to each individual channel respectively. Afterwards, the output was concatenated to one feature vector. The transformer was implemented using pyts (version 0.12.0)^49^. The computed features were passed into the same selection of classifiers as described above (see option A).

Option C integrates automatic feature extraction via DL. XceptionTime was originally developed to classify multichannel surface electromyography (sEMG) signals^27^. In this work, we connected four XceptionTime modules in series to capture temporal and spatial information of the signal. The model involves simultaneous analysis of multiple channels and different kernel sizes to address long- and short-time intervals. In early experiments, we found most stable results when increasing the time series length and reducing the number of channels. To do so, we kept the channels distinct by axes, sensor and arm, but concatenated signals from different assessment steps. The implementation is based on tsai (version 0.2.24)^50^. Balanced cross-entropy loss in combination with the 1-cycle learning rate policy^51^ was used for training. Since DL typically requires larger amounts of training data compared to traditional ML, data augmentation was applied during training. Similar to Um et. al, we performed randomised time warping and axes rotations^52^.

Since the questionnaire data is far simpler due to its tabular structure, only one classifier was used here. For option D we used CatBoost that is based on gradient-boosted decision trees and was developed to efficiently handle categorical variables^28^. We utilised the official Python implementation from the catboost package (version 1.0.3). The learning rate was set to the default adjustment according to dataset properties and number of iterations.

While our dataset is age-matched between classes, the cohorts are still imbalanced in terms of gender and class distributions. For potential confounding variables, it is important to balance their influence during training, for example by weighted bootstrapping, to prevent learning potential spurious correlations^53^. Resampling techniques, however, may introduce duplicate samples that could also increase the risk of overfitting, if not regularised with correction methods. As an alternative approach to this problem, we use sample weighting in all loss computations. We implemented an approach similar to the balanced class weighting from scikit-learn, which adjusts weights inversely proportional to class frequencies. In our case, we considered groups of similar gender and class for the respective weighting.

**Fig. 5:**
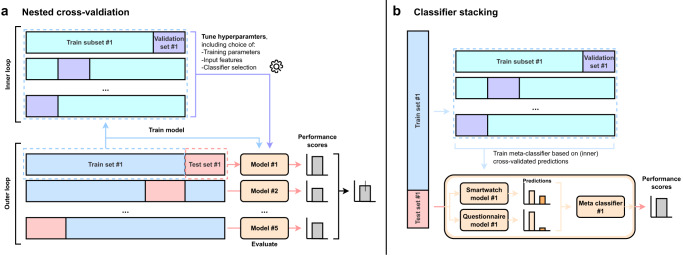
Overview of the nested cross-validation and classifier stacking. **a** Nested cross-validation (CV): A 5-fold CV separates the original dataset into a train set and a test set, referred to as the outer loop. Per fold, the selected train set is further separated into an internal train set and a validation set using an internal 5-fold CV, referred to as the inner loop. Using this inner CV, we perform a hyperparameter optimisation, including the selection of input features, model architecture and training parameters, for each of the five train-test splits. We apply this approach both to the smartwatch data and to the questionnaire data separately. Per test set, we report the best classifier setup and the achieved performance scores. Additionally, an average score is computed over all test results. **b** Classifier stacking: The classifier stacking is used to combine the informational value of different data modalities that are the smartwatch and questionnaire data. Per test fold, both smartwatch and questionnaire data are passed to the stacking classifier that internally forwards the data to the model setup optimised for smartwatch data and the model setup optimised for questionnaire input respectively (see Table 1). The prediction of each of these internal classifiers based on the internal CV are used to fit a logistic regression model, referred to as the meta-classifier. Per outer test fold, the final classification is performed by passing the predictions of the subclassifiers trained on the complete train fold to the meta-classifier.

Due to the multi-modal nature of the captured data (high-resolution time series data by smartwatches and tabular data by symptom questionnaires), additionally a combinatory ML approach was utilised via classifier stacking^54^. By doing so, different ML sub-models are trained and a final meta-classifier will be systematically trained and tested via CV (Fig. 5). After the internal optimisation of the individual classifiers per fold, we combined the strength of these predictors using our ML ensemble. To effectively utilise the two data modalities, we performed a modified version of classifier stacking where data from different sources are passed to different sub-classifiers respectively. Per test fold, we used the best model setup for smartwatch data and the best model setup for questionnaire input from the previous optimisation via the internal CV. A logistic regression model was applied as final meta-classifier on top of the individual sub-classifiers to consider all data modalities and compute the final label. During training, internal CV was used so that this final classifier was fitted on cross-validated probabilistic predictions of the sub-classifiers to increase generalisation^55^. The final classification is performed by passing the predictions of the subclassifiers trained on the complete train fold to the meta-classifier.

### Reporting summary

Further information on research design is available in the Nature Research Reporting Summary linked to this article.

### Supplementary information

Reporting summary

## Data Availability

The data presented in this study—the PADS (Parkinson’s Disease Smartwatch) dataset will be publicly available with publication of this paper. The dataset includes all 469 individual assessments and detailed descriptions on how to work with the data. For all participant samples, we include signal data recorded from the two wrist-worn smartwatches during 11 assessment steps. Further, we include details on demographics, medical history, and the self-reported non-motor symptoms. Dataset link: https://uni-muenster.sciebo.de/s/q69vUfRc9vgBoWX.

## Code availability

The Python scripts used for the ML analysis are made publicly available. Additionally, we include hyperparameters and randomisation seeds for the CV that were used in our presented experiments. Code link: https://imigitlab.uni-muenster.de/published/pads-project.
