# Author Correction: Machine Learning in the Parkinson’s disease smartwatch (PADS) dataset

**DOI:** 10.1038/s41531-024-00710-5

**Published:** 2024-04-28

**Authors:** Julian Varghese, Alexander Brenner, Michael Fujarski, Catharina Marie van Alen, Lucas Plagwitz, Tobias Warnecke

**Affiliations:** 1 Institute of Medical Informatics, University of Münster, Münster, Germany; 2 European Research Centre of Information Systems, University of Münster, Münster, Germany; 3 Department of Neurology and Neurorehabilitation, Klinikum Osnabrück - Academic teaching hospital of the University of Münster, Osnabrück, Germany

**Keywords:** Diagnostic markers, Databases, Parkinson's disease

Correction to: *npj Parkinson’s Disease* 10.1038/s41531-023-00625-7, published online 05 January 2024

In this article the wrong figure appeared as Fig. 3; the figure should have appeared as shown below.
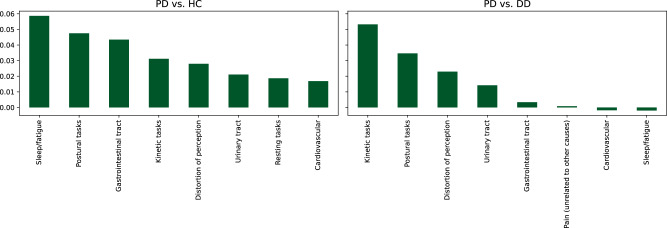


In the Results subsection ‘Feature importance analysis’ (p5) the following sentence was incorrectly given: “The “Sexual function” questions rank highest in the classification task PD vs. HC, while this feature group did not appear in the top ten for PD vs. DD. This indicates that the feature group is useful in distinguishing healthy samples from pathological ones, but may not be PD specific.” This should have been: “The “Sleep/fatigue” questions rank highest in the classification task PD vs. HC, while this feature group has marginal influence for PD vs. DD. This indicates that the feature group is useful in distinguishing healthy samples from pathological ones, but may not be PD specific.”

The pdf and the original article have been corrected.

