# Supplementary material for: Machine Learning in the Parkinson’s disease smartwatch (PADS) dataset
Source: NPJ Parkinsons Dis. 2024 Jan 5;10:9. doi: 10.1038/s41531-023-00625-7 (PMC10770131; doi:10.1038/s41531-023-00625-7)
Supplement: Supplementary file 1 — Reporting summary [file 41531_2023_625_MOESM1_ESM.pdf]

nature portfolio

Corresponding author(s): Julian Varghese
Last updated by author(s): Dec 15, 2023

Reporting Summary

Nature Portfolio wishes to improve the reproducibility of the work that we publish. This form provides structure for consistency and transparency in reporting. For further information on Nature Portfolio policies, see our Editorial Policies and the Editorial Policy Checklist.

Statistics

For all statistical analyses, confirm that the following items are present in the figure legend, table legend, main text, or Methods section.

n/a	Confirmed	
☐	☒	The exact sample size (*n*) for each experimental group/condition, given as a discrete number and unit of measurement
☐	☒	A statement on whether measurements were taken from distinct samples or whether the same sample was measured repeatedly
☒	☐	The statistical test(s) used AND whether they are one- or two-sided<br>*Only common tests should be described solely by name; describe more complex techniques in the Methods section.*
☒	☐	A description of all covariates tested
☒	☐	A description of any assumptions or corrections, such as tests of normality and adjustment for multiple comparisons
☐	☒	A full description of the statistical parameters including central tendency (e.g. means) or other basic estimates (e.g. regression coefficient) AND variation (e.g. standard deviation) or associated estimates of uncertainty (e.g. confidence intervals)
☒	☐	For null hypothesis testing, the test statistic (e.g. *F*, *t*, *r*) with confidence intervals, effect sizes, degrees of freedom and *P* value noted<br>*Give P values as exact values whenever suitable.*
☒	☐	For Bayesian analysis, information on the choice of priors and Markov chain Monte Carlo settings
☒	☐	For hierarchical and complex designs, identification of the appropriate level for tests and full reporting of outcomes
☒	☐	Estimates of effect sizes (e.g. Cohen's *d*, Pearson's *r*), indicating how they were calculated

Our web collection on statistics for biologists contains articles on many of the points above.

Software and code

Policy information about availability of computer code

Data collection	For data collection a custom application integrating smart consumer devices and a centralised database, which we refer to as the Smart Device System (SDS), has been used.
Data analysis	Data analysis was performed with Python using the libraries scikit-learn (version 1.1.0), pytorch (version 1.11.0), and skorch (version 0.11.0). Custom code is made available, see the code availability statement.

For manuscripts utilizing custom algorithms or software that are central to the research but not yet described in published literature, software must be made available to editors and reviewers. We strongly encourage code deposition in a community repository (e.g. GitHub). See the Nature Portfolio guidelines for submitting code & software for further information.

Data

Policy information about availability of data

All manuscripts must include a data availability statement. This statement should provide the following information, where applicable:

- Accession codes, unique identifiers, or web links for publicly available datasets
- A description of any restrictions on data availability
- For clinical datasets or third party data, please ensure that the statement adheres to our policy

The data presented in this study – the PADS (Parkinson's Disease Smartwatch) dataset will be publicly available with publication of this paper. The dataset includes

all 469 individual assessments and detailed descriptions on how to work with the data. For all participant samples, we include raw signal data recorded from the two wrist-worn smartwatches during 11 assessment steps. Further, we include details on demographics, medical history, and the self-reported non-motor symptoms.

Research involving human participants, their data, or biological material

Policy information about studies with human participants or human data. See also policy information about sex, gender (identity/presentation), and sexual orientation and race, ethnicity and racism.

Reporting on sex and gender	Gender information were collected via self-completed electronic questionnaires after written informed consent.
Reporting on race, ethnicity, or other socially relevant groupings	The collected data in our study that directly relates to socially relevant groups are formed by age and gender. To control for confounding effects, the complete dataset has been age-matched. While the dataset still was imbalanced in terms of gender distribution, which is also due to the higher prevalence of PD in males, we additonally applied two methods to control for potential biases. First, the training of all classifiers were performed with sample weights that were balanced in terms of class and gender. Second, we extracted a subset of our dataset that was additionally matched by gender. Final results are additionally reported using only the matched subsets of each test fold.
Population characteristics	The relevant population characteristics are: gender, age, age at diagnosis (if applicable), height, weight.
Recruitment	All sessions were recorded upon routine visits, including accompanying persons, to the outpatient clinic of movement disorders at the University Hospital Münster in Germany, which is one of the largest PD assessment centres in Germany.
Ethics oversight	The prospective study recruited participants from the year 2018 until 2021. It has been registered on ClinicalTrials.gov with the ID NCT03638479 (registration date 20-08-2018) and approved by the ethical board of the University of Münster and the physician's chamber of Westphalia-Lippe (Reference number: 2018-328-f-S). All participants gave written informed consent to take part. The study design and the protocol have been published previously

Note that full information on the approval of the study protocol must also be provided in the manuscript.

Field-specific reporting

Please select the one below that is the best fit for your research. If you are not sure, read the appropriate sections before making your selection.

☒ Life sciences ☐ Behavioural & social sciences ☐ Ecological, evolutionary & environmental sciences

For a reference copy of the document with all sections, see nature.com/documents/nr-reporting-summary-flat.pdf

Life sciences study design

All studies must disclose on these points even when the disclosure is negative.

Sample size	To our knowledge, proper sample size estimation could not be performed, since it cannot be infered how many samples the machine learning model will require. However, we estimated the sample size using basic assumptions of machine learning principles, see the previous publication of the study design: https://doi.org/10.3389/fneur.2019.00048
Data exclusions	Quality, correctness, and completeness of diagnoses and raw data were checked throughout the study and after completion. Erroneous records and samples with missing data were removed (two samples dropped out due to missing data; from the youngest controls 33 were randomly removed as they did not match the age distribution of PD patients).
Replication	All experiment code and model check points are made publicly available. Results can be replicated by re-running the scripts, which has been tested after cloning the code repository.
Randomization	No randomization performed.
Blinding	No blinding performed.

Reporting for specific materials, systems and methods

We require information from authors about some types of materials, experimental systems and methods used in many studies. Here, indicate whether each material, system or method listed is relevant to your study. If you are not sure if a list item applies to your research, read the appropriate section before selecting a response.

Materials & experimental systems

n/a	Involved in the study
☒	☐ Antibodies
☒	☐ Eukaryotic cell lines
☒	☐ Palaeontology and archaeology
☒	☐ Animals and other organisms
☐	☒ Clinical data
☒	☐ Dual use research of concern
☒	☐ Plants

Methods

n/a	Involved in the study
☒	☐ ChIP-seq
☒	☐ Flow cytometry
☒	☐ MRI-based neuroimaging

Clinical data

Policy information about clinical studies

All manuscripts should comply with the ICMJE guidelines for publication of clinical research and a completed CONSORT checklist must be included with all submissions.

Clinical trial registration: NCT03638479

Study protocol: The study protocol has been published previously: https://doi.org/10.3389/fneur.2019.00048

Data collection: All sessions were recorded upon routine visits to the outpatient clinic of movement disorders at the University Hospital Münster in Germany, which is one of the largest PD assessment centres in Germany. In the three-year study, three participant groups were recorded: 1) Parkinson's Disease, 2) differential diagnoses, and 3) healthy controls.

Outcomes: The primary study goal was to enable a novel research dataset with two-sided smartwatch-based sensor measures from interactive neurological assessments, non-motor symptoms questionnaires and information about clinical history. With the publiction of the dataset, hodling 469 individual subjects, the goal has been achieved. Further, as a secondary goal, the diagnostic accuracy of the system has been evaluated using a wide range of machine learning methods with a nested-cross validation approach. The models achieved an average balanced accuracy of 91.16% in the classification Parkinson's Disease vs. healthy controls, while Parkinson's Disease vs. differential diagnoses scored 72.42%

Plants

Seed stocks: *Report on the source of all seed stocks or other plant material used. If applicable, state the seed stock centre and catalogue number. If plant specimens were collected from the field, describe the collection location, date and sampling procedures.*

Novel plant genotypes: *Describe the methods by which all novel plant genotypes were produced. This includes those generated by transgenic approaches, gene editing, chemical/radiation-based mutagenesis and hybridization. For transgenic lines, describe the transformation method, the number of independent lines analyzed and the generation upon which experiments were performed. For gene-edited lines, describe the editor used, the endogenous sequence targeted for editing, the targeting guide RNA sequence (if applicable) and how the editor was applied.*

Authentication: *Describe any authentication procedures for each seed stock used or novel genotype generated. Describe any experiments used to assess the effect of a mutation and, where applicable, how potential secondary effects (e.g. second site T-DNA insertions, mosiacism, off-target gene editing) were examined.*
